# The Measure Matters: An Investigation of Evaluative and Experience-Based Measures of Wellbeing in Time Use Data

**DOI:** 10.1007/s11205-016-1429-8

**Published:** 2016-08-20

**Authors:** Paul Dolan, Laura Kudrna, Arthur Stone

**Affiliations:** 1 Department of Social Policy, London School of Economics and Political Science, Houghton Street, London, WC2A 2AE UK; 2 Center for Self-Report Science, University of Southern California Dornsife, 3620 McClintock Ave., Los Angeles, CA 90089-1061 USA

**Keywords:** Happiness, Purpose, Unemployment, Time use, Measurement

## Abstract

Measures of subjective wellbeing (SWB) are used to understand how people think and feel about their lives and experiences. But the measure used matters to conclusions about how well people’s lives are going. This research compares life evaluations and experienced SWB using nationally representative time use diaries, advancing previous research because diaries are less subject to recall biases than other, more popular methods. Analyses of over 20,000 US residents in 2012–2013 show life evaluations are more closely associated with positive and negative affect than experienced meaningfulness. Women have higher SWB than men except for negative affect, older age groups have higher SWB than middle age groups except for experienced meaningfulness, and younger age groups report the lowest experienced meaning. The unemployed have low life evaluations but experiences of SWB are similar across employment groups. A complete picture of SWB requires a complete set of measures.

## Introduction

Subjective wellbeing (SWB) is a broad term for how people think about their lives and their everyday feelings (Diener et al. 2003). There have been some attempts to classify the dimensions and measures of SWB (Kahneman and Riis 2005; NAS 2014; OECD 2013; Pavot and Diener 1993b; Ryff and Keyes 1995). A widely recognized distinction is between “evaluations”, how people think about their lives, and “experiences”, how people feel on more of a day-to-day basis (Pavot and Diener 1993b; Kahneman and Riis 2005). SWB is captured in various ways within these categories. For evaluations, people are often asked how satisfied they are with their life overall, or with specific life domains, such as work or relationships (Dolan et al. 2008; Pavot and Diener 1993a). They may also be asked whether their lives have meaning, or if they consider them worthwhile, which is often referred to as ‘eudemonic’ SWB (Aristotle 2002; Ryff and Keyes 1995).

Experiences of SWB are typically collected using three methods. The most direct is experience sampling (ESM) or Ecological Momentary Assessment (EMA), where people rate how they feel at the time they are asked, e.g. ‘how happy do you feel right now?’ This method is least subject to recall biases because data are collected ‘in-the-moment’ (Scollon et al. 2003). In the Day Reconstruction Method (DRM) people fill out a diary of yesterday’s activities and rate their feelings during them (Kahneman et al. 2004). The third approach is to use a single-item indicator asking people how they felt over a certain time period e.g. ‘how happy did you feel today (or yesterday)?’ (ONS 2011). Longer recall periods reflect increasing degrees of evaluations of experiences. Various types of feelings are collected using these methods, usually in the form of positive and negative affect—‘hedonic’ or ‘experiential’ SWB—like joy, pain, stress, or worry, and some studies also ask about eudemonic feelings of meaning (Bradburn 1969; Christodoulou et al. 2014; White and Dolan 2009).

These kinds of distinction are often overlooked but they matter because it is possible to have high (low) SWB on one measure and low (high) SWB on another. The determinants of SWB may also differ according to the measure used (Kahneman and Deaton 2010; Luhmann et al. 2012; NAS 2014; OECD 2013). Employed people are more satisfied with their lives than the unemployed, for example, yet their daily experiences are similar in DRM data (Knabe et al. 2010). People who earn more than $75,000 annually are increasingly more satisfied with their lives but they do not have higher SWB according to single-item measures of hedonic experience (Kahneman and Deaton 2010). Middle age is a less satisfying time of life according to evaluative measures of SWB, but some studies suggest that middle aged people have higher experiential SWB than younger people, although there is mixed evidence in this area (Blanchflower and Oswald 2008b; Carstensen et al. 2011; Stone et al. 2010). There are differences within evaluations and experiences, too, such as the finding that higher income is not associated with daily experiences of happiness but it is with less sadness (Kushlev et al. 2015).

There are three major limitations to prior research. First, much of our understanding of differences between SWB measures comes from comparing studies using one subset of measures with those using another subset. In these cases, the relative effects of different determinants may be attributable to differing sample characteristics. Second, where within-sample comparisons are made, evaluations and experiences of SWB are often contrasted with single-item measures of experiential of SWB over a particular duration, such as yesterday as in the Gallup survey (Kahneman and Deaton 2010; Stone et al. 2010). These evaluations of yesterday may be different to experiences as captured by ESM/EMA or DRM studies. Third, no studies have investigated eudemonic experiences alongside evaluations of life. We do not know, for example, if those who find their experiences to be meaningful are also those that evaluate their life positively.

A new survey of over 22,000 US residents conducted by the US Bureau of Labor Statistics allows us to address all three limitations. These data, from the 2012–2013 American Time Use Survey (ATUS), enable us to investigate the relationship between the various dimensions of SWB and their different determinants. It is the largest ever sample containing a measure of how people think about their life overall alongside measures of activity-based experiential SWB.

The evaluative measure is the Cantril Ladder, which asks people to rank their life relative to the best and worst possible lives. The hedonic experience-based measures are reports of feelings of happiness, stress, tiredness, sadness and pain during activities. The measure of eudemonic experience is the rating of how meaningful the activity was. Note that this represents a conceptual shift from how eudemonia is typically considered in the wellbeing literature. Rather than conceiving of meaning as a trait component of SWB (Ryff and Keyes 1995), this research considers meaning as a component of people’s daily experiences. This is another important and potentially different aspect of SWB (Biswas-Diener et al. 2009; NAS 2014), although currently there currently exists no research elucidating how meaning as a trait relates to meaning as a component of daily experiences. Our research will not address this issue directly, but it will provide new insights into the relationship between meaning as a daily experience with life evaluations, other aspects of daily experiences, and personal characteristics.

Of course, more measures of SWB would be preferable here, especially to complement the single-item measures of experienced pleasure and meaning, and evaluative SWB, to more fully capture these concepts (Hox 1997; Watson and Tellegen 1985). Unfortunately, we are limited by the measures available in ATUS.

In considering the determinants of SWB, we first focus on labour force status. Unemployment is of interest because of its consistently large, negative impact on evaluative SWB, and other SWB determinants are often compared to its effect to characterise their magnitude (Blanchflower and Oswald 2008b; Deaton 2011). It may be that these comparisons are dependent on the component of SWB assessed, and we seek to highlight whether this is the case. We additionally consider the relationship between SWB and the exogenous characteristics age, ethnicity and gender. These characteristics are chosen because self-selection cannot confound any causal interpretation of the results.

## Materials and Methods

### Sample

This study analysed the 2012–2013 American Time Use Survey wellbeing modules, which is a sample of 21,736 US residents aged 15 years+. Each respondent was interviewed in 2012 or 2013. A telephone interviewer contacted each respondent, collecting information using a computer assisted interview procedure (CATI) about what activities the respondent engaged in from 4a.m. the prior day until 4a.m. the current day. Details are available on about 390 activities such as work and socialising. From each diary, three activities were randomly selected regarding which the respondent provided SWB ratings. Note, however, that an error in the random selection meant the last daily activity was never chosen. The survey weights adjust for this, as mentioned in ‘Weighting’ below. Only two activities were rated by 512 people and only one by 30 people. In total, 64,636 activities were rated.

### Items

The exact wording of the experiential SWB questions was, “Please use a scale from 0 to 6, where a 0 means you did not experience this feeling at all and a 6 means the feeling was very strong. From 0 to 6, where a 0 means you were not [EMOTION] at all and a 6 means you were very [EMOTION], how [EMOTION] did you feel during this time?” The emotions were happy, tired, stressed, and sad. For pain the exact item was “how much pain did you feel during this time if any?”, and for meaning it was “how meaningful did you consider what you were doing?”, again with 0–6 scale response options. Previous research has established that negative and positive affect are separate components of experiential SWB and we treat them as such (Watson and Tellegen 1985). The measure of negative affect was the average of tired, stressed, sad and pain, while positive affect was measured with happiness.

The exact wording of the Cantril Ladder was, “Please imagine a ladder with steps numbered from 0 at the bottom to 10 at the top. The top of the ladder represents the best possible life for you and the bottom of the ladder represents the worst possible life for you. If the top step is 10 and the bottom step is 0, on which step of the ladder do you feel you personally stand at the present time?” This item was transformed in analyses where the ladder was an outcome variable by dividing it by one and two thirds such that it had a maximum of six. This was done in order to facilitate comparisons with the other experienced SWB measures that also have a maximum of six. Less than 1 % of each SWB measure contained missing information and these observations were excluded from the analyses.

Three categories of employment were created with ‘employed-at work’ and ‘employed-absent’ constituting the employed category, ‘unemployed-on layoff’ and ‘unemployed-looking’ the unemployed category, and not in labour force the final category. Fourteen categories of age with 4 years in each group were created in order to maintain a sufficient sample size in each group, as well as to illustrate SWB differences between groups that could be masked by summarising the relationship with age and SWB by fitting a linear or non-linear model to the continuous age variable. The smallest proportion of responses was in the 75–79 years group (3.29 %; weighted; 823 respondents) and the largest in the 50–54 years group (8.96 %; weighted; 1934 respondents). Gender and ethnicity were self-report variables. Only information on male or female was available for gender. The category ‘White only’ represented the White ethnic group and all other ethnic groups were combined into the Black and Minority Ethnic (BME) group.

### Analysis

The main analytic method was multiple linear regressions using the ordinary least squares (OLS) method of estimation. This method was chosen for its ease of interpretability and based on prior research suggesting that results of analyses of the determinants of SWB do not differ substantively if SWB items are treated as ratio rather than ordinal-level variables (Ferrer-i-Carbonell and Frijters 2004). Standard errors were clustered at the individual level, and these data were analysed at the activity level rather than the person level.

### Weighting

All analyses used survey weights. There are two types of survey weights in ATUS: activity weights, for activity-level data, and final weights, for respondent-level data. For analyses of the experiential SWB items, activity weights were used because these vary by activity, and for analyses of the Cantril Ladder, final weights were used because the Ladder only varies by individual. Analyses including both experiential SWB items and the Cantril Ladder were conducted with both sets of weights with results from the activity weights reported along with any substantive differences when using the final weights. Both the activity and final weights adjust for several aspects of the complex survey design, e.g. oversampling of certain demographic groups and unit nonresponse. The activity weights also adjust for the time respondents spent in each activity. Both sets of weights adjust for the error in the random selection of activities mentioned above.

### Controls

To preclude any interpretation that the bivariate relationships of labour force status, age, ethnicity or gender with SWB are causal, and suggest the extent to which other factors might account for them, controls were included in some models. To maintain comparability across models, the same controls were used regardless of whether the investigation was of life evaluation or experience-based SWB. To avoid overcontrolling, three models were tested: one without controls, one with limited “set one” controls, and one with full “set two” controls. For example, for the unemployment models, the following models were tested:

1 $$SWB_{a} = a + \beta \;Unemployment_{a} + e_{a}$$

2 $$SWB_{a} = a + \beta \;Unemployment_{a} + \beta \;Set\;one\;controls_{a} + e_{a}$$

3 $$SWB_{a} = a + \beta \;Unemployment_{a} + \beta \;Set\;two\;controls_{a } + e_{a}$$

where *a* is the activity and *e* is the error term.

The regression control variables were selected based on a review identifying the determinants of life evaluation (Dolan et al. 2008), which is associated with experienced SWB (Pavot and Diener 1993b). These were gender, ethnicity, marital and labour force status, education, self-rated health, time spent in religious activities during the diary day, hypertension (Blanchflower and Oswald 2008a), whether they had children, and household income. Telephone ownership was also included to proxy material deprivation (Dolan et al. 2008). Further controls associated specifically with experiential SWB were included: whether they felt rested (Tempesta et al. 2010) and amount of time spent alone (Oerlemans et al. 2011) on the diary day. To improve generalisability, typicality of days’ feelings was also included. Although the survey weights adjust for the proportion of weekdays and weekends sampled, they do not adjust for how typical the participants’ affect was on these days, and this variable may help to do so. Whether the participants took pain medicine on the diary day was also included as an additional indicator of health in addition to self-rated health and hypertension.

The limited “set one” controls were gender, ethnicity, marital and labour force status, and education. The full “set two” controls included the set one controls plus self-rated health, time spent doing religious activities, hypertension, whether they had children, household income, telephone ownership, whether felt rested, amount of time spent alone, typicality of days’ feelings, and whether they took pain medicine. Wave was also included with set one and two controls. Variance inflation factor tests never returned a value higher than six, indicating no significant multicollinearity among the predictors.

## Results

In the weighted estimates, 51.69 % were female, 52.18 % earned $50 K+ annually, and 51.77 % were married, with a mean age of 45.08 and a standard deviation of 18.57 years. 6.05 % were unemployed, 60.8 % were employed, and 33.15 % were not in the labour force. All SWB measures range from zero to six except for the Cantril Ladder, which ranges from zero to ten, and their means and standard deviations are presented in Table 1.

**Table 1:** Means, standard deviations, and ranges for each wellbeing measure (weighted)

	Mean	sd	N (individuals)
Happy	4.34	1.6	64,206
Stress	1.45	1.8	64,473
Tired	2.29	1.93	64,450
Sad	0.61	1.33	64,417
Pain	0.97	1.65	64,493
Negative affect*	1.33	1.19	64,185
Meaning	4.31	1.85	63,898
Cantril Ladder	7.13	2.02	64,636

* Average of stress, tired, sad and pain

### Relationship Between the Cantril Ladder and Experience-Based SWB

**Fig. 1:**
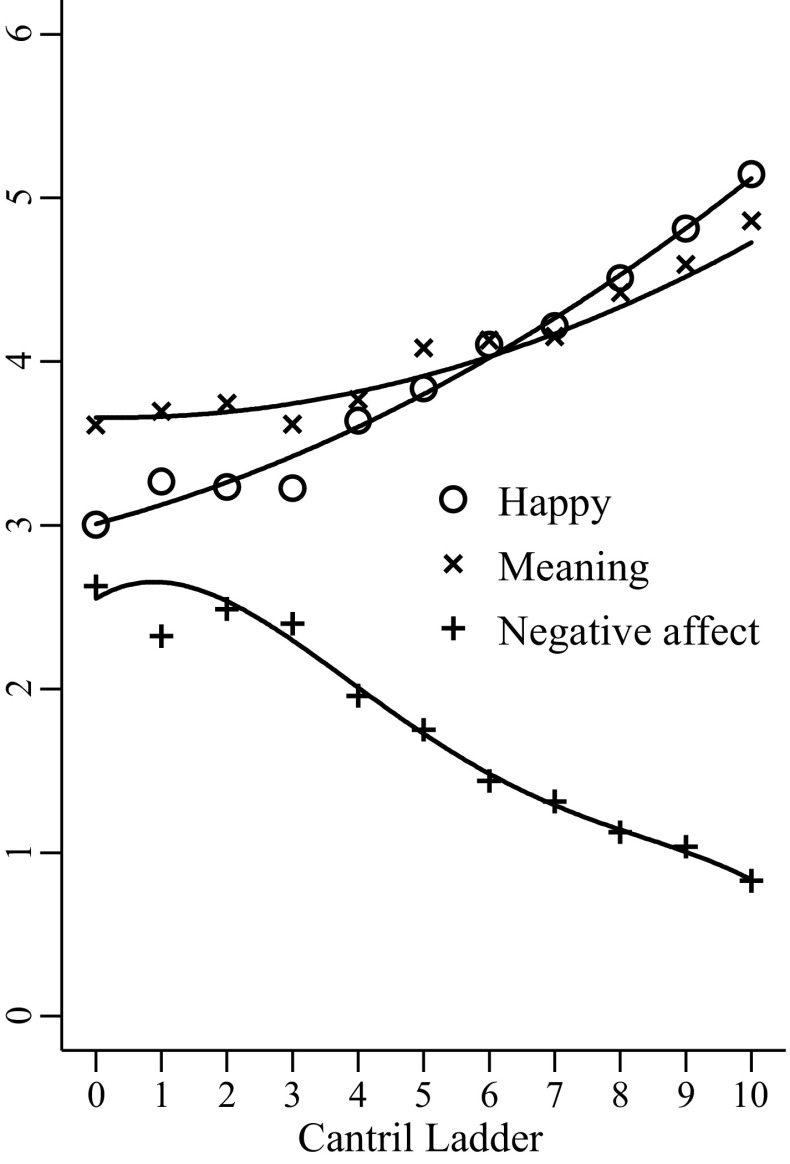
Average predicted values and lines of best fit for happy, negative affect and meaning at each level of the Cantril Ladder (without controls, activity weights)

In what follows, uncontrolled results are presented unless otherwise stated, and all instances where results differ with controls are discussed. The relationship of the Ladder with happiness, negative affect and meaning is shown in Fig. 1 and Appendix Table 2. This figure displays average predicted values for experience-based SWB measures at each level of the Ladder (from unadjusted OLS regressions, where experience-based SWB measures are the outcome variables and the Ladder is a categorical explanatory variable). Lines of best fit are also shown, where polynomial fits up to the power of nine were tested, and the highest statistically and practically significant polynomials are reported.

**Table 2:** Results of regressions explaining variance in happy, negative affect and meaning from the Cantril Ladder (without controls, activity weights)

Cantril Ladder	Happy			Negative affect			Meaning		
	b	se	*p*	b	se	*p*	b	se	*p*
0	Ref			Ref			Ref		
1	0.26	0.36	0.47	−0.31	0.31	0.33	0.08	0.39	0.83
2	0.23	0.29	0.43	−0.14	0.25	0.58	0.13	0.30	0.66
3	0.22	0.27	0.42	−0.23	0.23	0.32	0.00	0.28	0.99
4	0.63	0.27	0.02	−0.67	0.22	0.00	0.15	0.27	0.57
5	0.83	0.26	0.00	−0.88	0.21	0.00	0.47	0.26	0.08
6	1.10	0.26	0.00	−1.19	0.21	0.00	0.52	0.26	0.05
7	1.21	0.26	0.00	−1.31	0.21	0.00	0.54	0.26	0.04
8	1.51	0.26	0.00	−1.50	0.21	0.00	0.81	0.26	0.00
9	1.81	0.26	0.00	−1.59	0.21	0.00	0.98	0.26	0.00
10	2.14	0.26	0.00	−1.80	0.21	0.00	1.25	0.26	0.00
Constant	3.00	0.26	0.00	2.63	0.21	0.00	3.61	0.26	0.00

The Ladder is similarly associated with happiness (r2 = 0.09) and negative affect (r2 = 0.11) and less so with meaning (r2 = 0.03). In general, increasing Ladder scores are associated with increasing happiness and meaning and decreasing negative affect. For happiness, Ladder levels four and higher are significantly different from zero without and with controls; with the final weights, levels two and higher are different from zero without and with controls (see Fig. 1 for further discussion of these results). For negative affect, only levels four and higher of the Cantril Ladder are significantly lower than level zero without and with controls. For meaning, only Ladder levels six or higher are significantly higher than zero without controls, and with controls level five is also significantly higher than level zero.

**Fig. 2:**
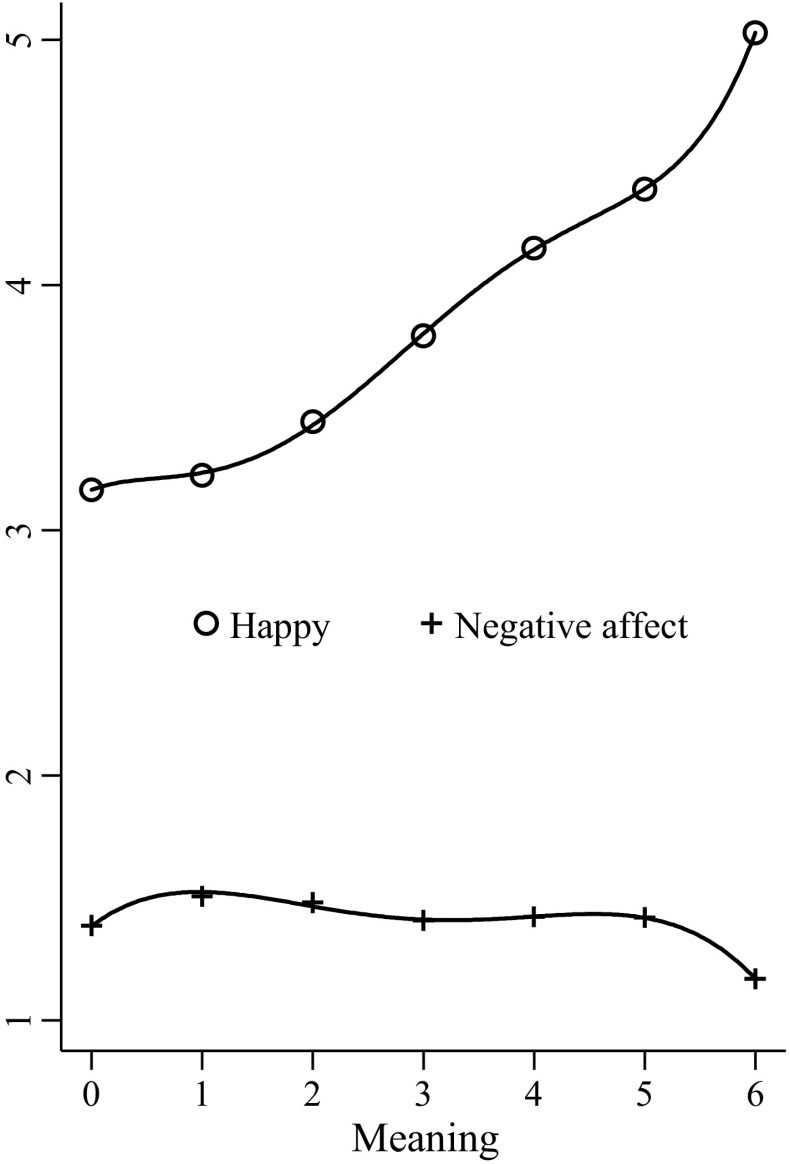
Average predicted values and lines of best fit for happy and negative affect at each level of meaning (without controls, activity weights)

Given the lack of research into experienced meaning relative to positive and negative affect, we also consider the relationship of meaning with positive and negative affect. Similar to Fig. 1, Fig. 2 displays average predicted values for positive and negative affect at each level of meaning (from unadjusted OLS regressions with meaning as the explanatory variable and happiness or negative affect as the outcome variable; see also Appendix Table 3). Meaning is more closely associated with positive affect (r2 = 0.16) than negative affect (r2 = 0.01). Meaning is positively associated with positive affect, with meaning levels two and higher always associated with more positive affect than level zero (see Fig. 2 for further discussion). Meaning is not strongly or consistently related to negative affect. Without controls, meaning level six is associated with significantly lower negative affect than meaning level zero, and when introducing controls, levels two through five are associated with significantly higher affect.

**Table 3:** Results of regressions explaining variance in happy and negative affect from meaning (without controls, activity weights)

Meaning	Happy			Negative affect		
	b	se	*p*	b	se	*p*
0	Ref			Ref		
1	0.06	0.10	0.55	0.12	0.07	0.08
2	0.28	0.09	0.00	0.09	0.06	0.10
3	0.63	0.08	0.00	0.02	0.05	0.68
4	0.99	0.08	0.00	0.04	0.05	0.47
5	1.23	0.08	0.00	0.03	0.05	0.49
6	1.86	0.08	0.00	−0.22	0.04	0.00
Constant	3.16	0.07	0.00	1.39	0.04	1.31

For happy, levels four and higher of the Cantril Ladder are significantly higher than level zero without and with controls (e.g. level four without controls, b = 0.63, se = 0.27, *p* < 0.05). With the final weights, however, levels two and higher were significantly different from zero without and with controls (e.g. level two without controls, b = 0.43, se = 0.22, *p* = 0.05). A quadratic ladder term fit the data best without controls (b2 = 0.01, se = 0.003, *p* < 0.01) and a sixth order polynomial was the best fit for all other models (e.g. final weights with full controls, b6 = −0.0001, se = 0.00005, *p* < 0.01).

For negative affect, only levels four and higher of the Cantril Ladder are significantly different from zero without and with controls (e.g. without controls level four, b = −0.67, se = 0.22, *p* < 0.001). A fourth order polynomial fit best without controls and with set two controls (b4 = −0.0007, se = 0.0003, *p* < 0.01 for both) and a seventh order polynomial fit best with set one controls (b7 = −0.00005, se = 0.00002, *p* < 0.01). Using the final weights, a seventh order polynomial fit best without controls (b7 = 0.00004, se = 0.0002, *p* < 0.05) and with set one controls (b7 = −0.00006, se = 0.0002, *p* < 0.01), and with set two controls a fourth order polynomial fit best (b4 = −0.0007, se = 0.0002, *p* < 0.01).

For meaning, only levels six and higher are significantly higher than level zero without controls (e.g. level six, b = 0.52, se = 0.26, *p* = 0.05); with set one and two controls, levels five and higher are significantly higher than level zero (e.g. level five with set one controls, b = 0.58, se = 0.26, *p* < 0.05). With the final weights, levels five and seven or higher were significantly different to level zero without controls (e.g. level five, b = 0.43, se = 0.21, *p* < 0.05); with sets one and two controls and the final weights, level six was additionally statistically significant (e.g. level six with set one controls, b = 0.59, se = 0.20, *p* < 0.01). An eighth order polynomial fit the data best without controls (b8 = 0.00004, se = 0.00002, *p* < 0.05) but it did not fit the data well at Ladder levels higher than eight. Thus the next best fit, a quadratic Ladder term, is shown (b2 = 0.01, se = 0.003, *p* < 0.01), which was also significant with set one controls (b2 = 0.006, se = 0.003, *p* = 0.05) but not set two controls (b2 = 0.005, se = 0.003, *p* > 0.05).

As indicated by the green line, higher levels of meaning are associated with increasingly higher levels of positive affect. Meaning levels two and higher are associated with significantly more positive affect than meaning level zero (e.g. level three, b = 0.63, se = 0.09, *p* < 0.01). A fifth order polynomial for meaning best fit the relationship with happiness (b5 = 0.003, se = 0.002, *p* < 0.05).

As indicated by the red line, meaning is not strongly or consistently related to negative affect. Only meaning level six is associated with significantly lower negative affect than meaning level zero (b = −0.22, se = 0.04, *p* < 0.001). With set two controls, meaning level six is still associated with lower negative affect than meaning level zero (b = −0.24, se = 0.04, *p* < 0.001) but meaning level two is associated with higher negative affect than meaning level zero (b = 0.12, se = 0.06, *p* < 0.05). With set two controls, all levels of meaning are associated with significantly higher negative affect than meaning level zero. These coefficients are all of similar magnitude and within each other’s confidence intervals (e.g. level four, b = 0.14, se = 0.04, *p* < 0.01). Meaning level six is no longer significantly different to meaning level zero with set two controls (b = −0.07, se = 0.04, *p* > 0.05). A fourth order polynomial for meaning best fit the relationship with happiness (b4 = −0.006, se = 0.002, *p* < 0.001).

### Relationships Between Labour Force Status and SWB

**Fig. 3:**
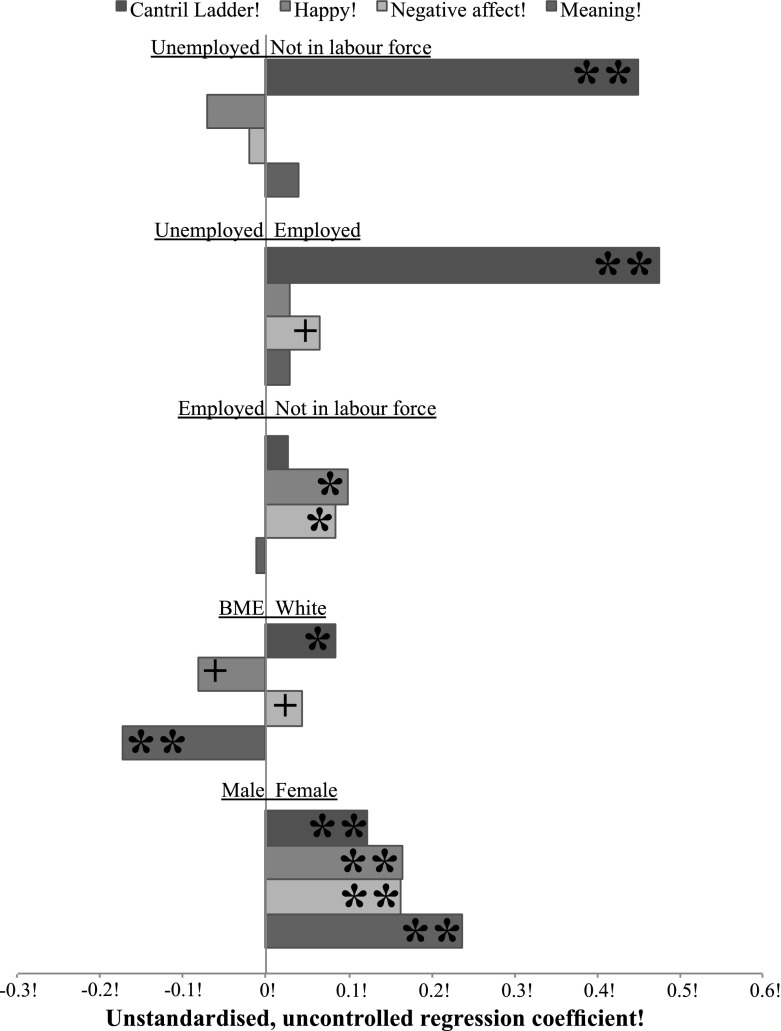
Plot of the unstandardised, uncontrolled regression coefficients for the relationship of labour force status, ethnicity and gender with SWB. *p < 0.01, **p < 0.001, *BME* black and minority ethnic, + = becomes significant with controls

In all further analyses, the Cantril Ladder was transformed to a scale ranging from zero to six (see Materials and Methods) ($\bar{x} = 4.28$, sd = 1.21). The relationship between unemployment and SWB is shown in Fig. 3. Looking at the top of Fig. 3, which compares people who are not in the labour force with the unemployed (the reference category), those not in the labour force have significantly higher Ladder scores than the unemployed (b = 0.48, se = 0.06, *p* < 0.001), while scores on all other measures do not significantly differ between these two groups. The employed also have higher Ladder scores than the unemployed (b = 0.45, se = 0.05, *p* < 0.001). The employed and those not in the labour force did not differ in their Ladder scores (b = 0.03, se = 0.02, *p* > 0.05). For happiness, negative affect, and meaning, the unemployed did not differ from the employed or from those not in the labour force (e.g. employed vs. unemployed for happiness (b = −0.08, se = 0.07, *p* > 0.05). When including set one controls, unemployed people had lower negative affect than those not in the labour force (b = −0.13, se = 0.06, *p* < 0.05), but all other set one and two control results were similar. Labour force status was similarly associated with the Ladder (r2 = 0.008) and negative affect (r2 = 0.001), less so with happiness (r2 = 0.0008), and the least so with meaning (r2 = <0.00001). The number of weeks of unemployment was never associated with SWB among the unemployed (*p* > 0.05).

### Relationships Between Age and SWB

**Fig. 4:**
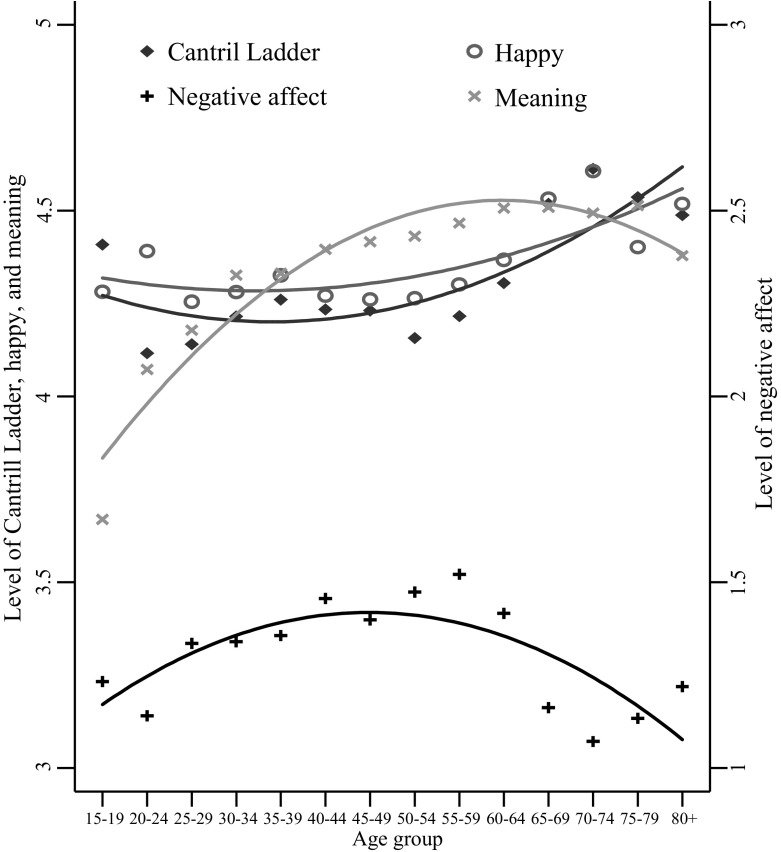
Average predicted values and lines of best fit for the Cantril Ladder, happy, negative affect and meaning by age group

The relationships between age and the various measures of SWB are shown in Fig. 4 and Appendix Table 4. This figure displays average predicted values of SWB at 4-year intervals of age, and lines of best fit for these values. Results are discussed relative to the middle age group, 50–54 year olds, based on prior research showing that middle-aged people have the lowest SWB (Dolan et al. 2008). Age was similarly associated with the Ladder (r2 = 0.01), negative affect (r2 = 0.01), and meaning (r2 = 0.02), and least closely associated with happiness (r2 = 0.004), with all relationships statistically significantly at the *p* < 0.001 level.

**Table 4:** Results of regressions explaining variance in the Cantril Ladder, happy, negative affect and meaning by age group

Cantril Ladder	Cantril Ladder			Happy			Negative affect			Meaning		
	b	se	*p*	b	se	*p*	b	se	*p*	b	se	*p*
15–19	0.25	0.05	0.00	0.02	0.07	0.81	−0.24	0.06	0.00	−0.76	0.10	0.00
20–24	−0.04	0.06	0.50	0.13	0.08	0.14	−0.33	0.06	0.00	−0.36	0.09	0.00
25–29	−0.02	0.05	0.75	−0.01	0.10	0.93	−0.14	0.06	0.02	−0.25	0.09	0.01
30–34	0.06	0.05	0.26	0.02	0.07	0.82	−0.13	0.06	0.02	−0.11	0.08	0.18
35–39	0.10	0.05	0.03	0.06	0.06	0.32	−0.12	0.05	0.02	−0.10	0.07	0.16
40–44	0.08	0.05	0.12	0.01	0.07	0.92	−0.02	0.06	0.75	−0.04	0.07	0.62
45–49	0.07	0.05	0.17	0.00	0.07	0.96	−0.07	0.06	0.19	−0.02	0.07	0.84
50–54	Ref			Ref			Ref			Ref		
55–59	0.06	0.05	0.26	0.04	0.07	0.60	0.05	0.06	0.45	0.03	0.07	0.64
60–64	0.15	0.05	0.00	0.10	0.07	0.14	−0.06	0.06	0.37	0.08	0.07	0.30
65–69	0.36	0.05	0.00	0.27	0.07	0.00	−0.31	0.06	0.00	0.08	0.08	0.31
70–74	0.46	0.06	0.00	0.34	0.09	0.00	−0.40	0.06	0.00	0.06	0.09	0.49
75–79	0.38	0.07	0.00	0.14	0.10	0.18	−0.34	0.07	0.00	0.08	0.11	0.43
80+	0.33	0.06	0.00	0.25	0.08	0.00	−0.25	0.07	0.00	−0.05	0.09	0.54
Constant	4.16	0.04	0.00	4.26	0.05	0.00	1.47	0.04	0.00	4.43	0.05	0.00

There is an overall positive quadratic relationship between age and the Ladder, and between age and happiness. For the Ladder, those aged 60+ years always had higher scores than those aged 50–54 years, whilst the younger ages (15–19 and 35–39 years) only had higher scores than 50–54 year olds without controls and with set one controls (15–19, 20–24, and 35–39 years). For happiness, only 65–69 year olds were always happier than 50–54 year olds across the uncontrolled and controlled results. There was not a clear pattern across uncontrolled and controlled results, with only ages 65–74 years having higher scores than those aged 50–54 years without controls; ages 20–24, 65–74 and 80+ having higher scores with set one controls; and with set two controls, 15–19 year olds were less happy than 50–54 year olds and only 65–69 and 70–74 year olds were happier.

An overall negative quadratic relationship is observed between age and negative affect, as well as between age and meaning. For negative affect, only those 65 years+ had significantly lower negative affect than 50–54 year olds across both uncontrolled and controlled results. With no controls, those younger than 40 years also had lower negative affect; with set one controls, those younger than 35 years also had lower negative affect; with set two controls, only those aged 20–24 years also had lower scores. For meaning, only the young had significantly higher scores than 50–54 year olds: with no controls, those younger than 30 years; with set one controls, those younger than 25 years; and with set two controls, those younger than 45 years.

From the figure, it would appear that most predicted values of the Cantril Ladder and happy are the same or higher than those for ages 50–54 years. In fact, for the Cantril Ladder, only age groups 15–19, 35–39 and 60 years and older had higher scores than those aged 50–54 years (e.g. 35–39, b = 0.10, se = 0.05, *p* < 0.05). The results also depend on the controls. With set one controls 20–24 year olds also had higher scores (b = 0.24, se = 0.06, *p* < 0.001) but 35–39 year olds did not (b = 0.056, se = 0.05, *p* > 0.05). With set two controls only those aged 60 years and higher had higher scores than 50–54 year olds (e.g. 60–64 years, b = 0.16, se = 0.05, *p* < 0.001). A positive quadratic age term fit the data best (b2 = 0.0002, se = 0.00003, *p* < 0.001).

For happiness, although many predicted values appeared higher than for those aged 50–54 years, only ages 65–74 years had significantly higher scores (b = 0.27, se = 0.07, *p* < 0.001 for 65–69 years; b = 0.34, se = 0.09, *p* < 0.001 for 70–74 years). The results again depend on the controls. With set one controls those aged 20–24 years also had higher happiness scores than 50–54 year olds (b = 0.30, se = 0.09, *p* < 0.01), as did those 80+ years (b = 0.25, se = 0.09, *p* < 0.01). With set two controls 15–19 year olds were less happy than 50–54 year olds (b = −0.31, se = 0.11, *p* < 0.01) and only 65–69 and 70–74 year olds were happier (b = 0.17, se = 0.07, *p* < 0.05 and b = 0.21, se = 0.09, *p* < 0.05, respectively). A positive quadratic age term fit best without controls (b2 = 0.0001, se = 0.00004, *p* < 0.001) but with set two controls the quadratic term was not significant (b2 = 0.00001, se = 0.00005, *p* > 0.05), although the linear term was (b = 0.004, se = 0.001, *p* < 0.01).

For negative affect, it would appear from the figure that most predicted values were the same or lower than for those aged 50–54 years, with the exception of those aged 55–59 years. In fact, only those younger than 40 years had significantly lower Ladder scores than those aged 50–54 years (e.g. 35–39 years, b = −0.12, se = 0.05, *p* < 0.05), as well as those aged 65+ years (e.g. 80+ years, b = −0.25, se = 0.07, *p* < 0.001). Other age groups did not differ but again, the results depend on the controls. With set one controls those aged 35–39 years no longer had significantly less negative affect than those aged 50–54 years (b = −0.12, se = 0.06, *p* > 0.05). With set two controls, only those aged 20–24 years (b = −0.13, se = 0.06, *p* < 0.05) and 65+ (e.g. 80+ years, b = −0.27, se = 0.06, *p* < 0.001) had lower scores than 50–54 year olds. A negative quadratic age term fit best (b2 = −0.0003, se = 0.00003, *p* < 0.001).

For meaning, it would appear from the figure that age groups older than 50–54 years had the same or higher SWB, whereas younger age groups had the same or lower SWB. In fact, only those younger than 30 years had lower scores than those aged 50–54 years (e.g. 25–29 years, b = −0.05, se = 0.09, *p* < 0.01) and the other groups did not significantly differ. Including controls affected which younger age groups were significantly different from those aged 50–54 years. Only those younger than 25 years had lower scores with set one controls (e.g. 20–24 years, b = −0.22, se = 0.10, *p* < 0.05). With set two controls, all those younger than 45 years experienced less meaning than those aged 50–54 years (e.g. 35–39 years, b = −0.22, se = 0.08, *p* < 0.01). A negative quadratic age term fit the data best (b2 = −0.0007, se = −0.00005, *p* < 0.001). Note that in some instances higher order polynomials greater than the reported quadratic fits were statistically significant, but they were never greater than eight millionths of one SWB unit, rendering them practically insignificant, thus they are not reported here.

### Relationship Between Ethnicity and Gender and SWB

The relationship of ethnicity and gender with SWB is shown in Fig. 2. Ethnicity was similarly associated with the Ladder (r2 = 0.0007), positive affect (r2 = 0.0004), and negative affect (r2 = 0.0002), and most closely with meaning (r2 = 0.001). BMEs had lower Ladder scores than Whites (b = −0.08, se = 0.03, *p* < 0.01), but not with set one (b = 0.01, se = 0.03, *p* > 0.05) or set two controls (b = 0.04, se = 0.03, *p* > 0.05). There was no difference in happiness between these ethnic groups without controls (b = 0.08, se = 0.05, *p* > 0.05), but BMEs were significantly happier than Whites with set one (b = 0.13, se = 0.05, *p* < 0.01) and two controls (b = 0.14, se = 0.05, *p* < 0.01). There was also no difference in negative affect between these groups without controls (b = −0.04, se = 0.03, *p* > 0.05), but BMEs had less negative affect than Whites with set one (b = −0.08, se = 0.03, *p* < 0.05) and two controls (b = −0.06, se = 0.03, *p* < 0.05). BMEs experienced significantly higher meaning (b = 0.17, se = 0.05, *p* < 0.001).

Gender was similarly associated with the Ladder (r2 = 0.003), positive (r2 = 0.003) and negative affect (r2 = 0.005), and meaning (r2 = 0.004). Women had higher Ladder scores than men (b = 0.12, se = 0.02, *p* < 0.001). Women were also happier (b = 0.16, se = 0.03, *p* < 0.001) and experienced more meaning (b = 0.24, se = 0.04, *p* < 0.001), but reported more negative affect (b = 0.16, se = 0.02, *p* < 0.001).

## Discussion

This research investigated how different components of SWB are related and considered which groups do well and badly on them. The results show that we cannot know how well people’s lives are going from a single component. Although evaluative and experiential SWB are related, the relationship is weak. Someone’s happiness tells us more about their Ladder scores than does how meaningful they consider their activities to be or how much negative affect they experienced during them. This is consistent with previous work establishing that there is an affective component to evaluations (Pavot and Diener 1993b), but that they are different constructs. The results also partly confirm the results of previous work showing a small correlation of life evaluation with positive and negative affect, but no correlation with daily (eudemonic) experiences of engagement (Kahneman et al. 2004; Kopperud and Vitters⊘ 2008; Pavot and Diener 1993a). Moreover, experienced purpose was only weakly associated with positive affect and (especially) negative affect. Thus, it may be important ask about eudemonic experiences in addition to other SWB measures in order to obtain a complete picture of SWB.

Examining the correlates of SWB also demonstrated the importance of separately considering the various components of SWB. Whether the unemployed had low SWB depended upon the measure. Consistent with most previous work (Dolan et al. 2008), the unemployed had lower life evaluations than the employed and those not in the labour force. But the unemployed generally did not differ in experienced SWB from other labour force groups. This result is consistent with Knabe et al. (2010), who showed the unemployed have similar hedonic wellbeing to the employed once accounting for hedonic duration. It is inconsistent with Luhmann et al.’s (2012) longitudinal review suggesting unemployment has lasting negative effects on experienced SWB, although the authors did note significant heterogeneity between studies’ results. Our results appear to confirm no relationship between unemployment and experiential SWB. They additionally suggest the lack of a relationship is not due to adaptation, as the duration of unemployment was not associated with SWB.

Which age groups are doing well also differed depending on the SWB measure, although the quadratic ‘U-shape’ observed in other research was also evidenced here across measures (Blanchflower and Oswald 2008b; Weiss et al. 2012). It was observed without and with controls, like some (Stone et al. 2010) but unlike other US research (Blanchflower and Oswald 2008b, 2009). According to life evaluation, happiness and negative affect, older age groups had robustly higher SWB than the middle aged but the SWB of the young did not consistently differ from the middle aged. In contrast, according to experienced meaning, older age groups did not differ from the middle aged, but the young reported less experienced meaning. So, we generally confirm prior research establishing that older age groups have better experiential SWB than the middle aged (Carstensen et al. 2011). Older age groups are doing well in their life evaluations and positive and negative affect, and younger age groups are doing badly in their of experiences of meaning. Notably, that the young experienced the least meaning relative to other ages in these data differs from prior research showing that the young experience more meaning in life than the middle aged (Steger et al. 2009). This suggests experiences of meaning may differ from evaluations of meaning.

The age results for happiness are somewhat similar to prior research using single-item measures of yesterday’s affect from the US Gallup survey (Stone et al. 2010). A quadratic U-shaped relationship emerged in both our and Gallup results, although younger age groups were less happy than the middle aged in Gallup, whereas they were not clearly so in ATUS. For negative affect in Gallup, separate rather than combined measures were used and there were differences across the measures. For example, stress decreased fairly linearly with age and was not U-shaped, while sadness was inversely U-shaped with age. Perhaps in Gallup an overall inverted U-shape would emerge for negative affect if the measures were combined as in this research. Disaggregating ATUS data suggests that stress decreases with age, as in Gallup, but in ATUS this decrease is less pronounced before 60 years than it is in Gallup. In ATUS, sadness also has an inverted U-shape, but the greatest amount of sadness occurs in the late 50 s and early 60 s rather than in the mid-50 s as in Gallup.

The SWB of ethnic groups also depended on the measure. Although Whites had higher life evaluations than BMEs, BMEs had better experiences: they were happier, experienced less negative affect and more meaning. This is consistent with previous life evaluation research (Dolan et al. 2008), although there is a lack of DRM research on experiential SWB for comparison. In nationally representative UK data, single measures of affect yesterday suggest BMEs also have worse experiences, and the differences with our results could be due either to sample or measure differences (Deeming 2013).

Women had higher SWB than men across every measure except negative affect, where they experienced more. Previous research is mixed on gender differences in life evaluation, although the predominate result is that men’s evaluations are higher, and so our results are in the minority (Dolan et al. 2008). In some DRM samples, positive affect does not vary by gender (Bakker et al. 2013; Oerlemans et al. 2011), whereas in others, women score higher in positive and negative affect than men (Knabe et al. 2010). Our results are consistent with the latter and suggest that women have a higher intensity of SWB regardless of whether it is evaluative or experiential. Interestingly, women do not appear to differ in happiness from men in other studies using single-item measures of yesterday’s affect (Deeming 2013; Stone et al. 2010).

There are three possible reasons why our results differ from those reported in Gallup. First, as mentioned, in ATUS levels of SWB were measured with episodic recall of yesterday whereas in Gallup full-day “yesterday” measures were employed. It has not, however, been empirically demonstrated that one measure is superior. Second, ATUS used a geographic sampling frame, whereas Gallup relied on random digit dialling, although both samples sought to minimize non-representativeness with weights.

Another possible reason for the discrepancies—usually in the direction of not detecting findings from Gallup in ATUS—could be the two assessment techniques’ reliability. While the original DRM obtained ratings of all episodes for a day, the ATUS wellbeing module sampled only three. Relative to the original DRM, ATUS estimates of experiential SWB should be less reliable given the absence of much of the day’s content. Perhaps this reduced our tests’ statistical power. Future research could emulate the power of three versus full episode measures of affect from DRM data, and also compare this to single-item measures of yesterday’s affect, informing this possible explanation.

In conclusion, the component of SWB assessed matters. We cannot know how well people’s lives are going by simply asking them to evaluate their life overall. People’s life evaluations are not strongly related to their experiences of SWB, especially when considering how meaningful their experiences are. From people’s life evaluations, we would conclude that unemployed people are doing worse than the employed, and that Whites are doing better than BME ethnic groups. But when considering these groups’ experiences, the conclusions are different: unemployed people fare no worse than the employed, and BME groups fare better than those of a White ethnicity.

The type of experience assessed matters, too. Older age groups would seem to be faring better than the middle aged according to positive and negative affect—and life evaluations—but older age groups are no different to the middle aged according to their experienced meaning. Women would appear to have higher SWB than men if we only asked about experienced happiness and meaning or life evaluations, but they would appear to have lower SWB than men if we only asked about negative affect.

A complete picture of SWB therefore requires an assessment of all components of SWB. Measures of SWB are increasingly being used in policymaking e.g. to allocate scarce resources (Fujiwara and Campbell 2011), and so this conclusion requires a deeper normative discussion about which type of SWB should be used in which contexts. Data of the kind presented here can never resolve the normative debate but they can inform it, providing evidence on the consequences of giving more or less weight to particular measures.

## Appendix

See Tables 2, 3, 4.
